# Combined Creatinine and Cystatin C Equations Improve Estimation of Glomerular Filtration Rate in Kidney Transplant Recipients

**DOI:** 10.3389/ti.2026.15529

**Published:** 2026-02-10

**Authors:** Etienne Mondésert, Anne-Sophie Bargnoux, Ilan Szwarc, Moglie Le Quintrec, Georges Mourad, Jean-Paul Cristol

**Affiliations:** 1 Department of Biochemistry, University Hospital of Montpellier, Montpellier, France; 2 PhyMedExp, University of Montpellier, INSERM, CNRS, Montpellier, France; 3 Department of Nephrology, University Hospital of Montpellier, Montpellier, France; 4 AIDER Santé Fondation Charles Mion, Montpellier, France

**Keywords:** chronic kidney disease epidemiology collaboration CT, creatinine, cystatin C, glomerular filtration rate, kidney transplantation

## Abstract

Glomerular filtration rate (GFR) is a crucial parameter in post-transplant follow-up (PTF). CKD-EPI 2009 creatinine-based equation remains the most used estimated GFR (eGFR) and only few data are available on the other equations, based on creatinine, cystatin C or their combination. We evaluated 10 GFR estimation equations on 242 kidney-transplant recipient patients having measured GFR (mGFR) determination (urinary clearance of ^99m^Tc-DTPA) with simultaneous plasma enzymatic creatinine and serum cystatin C (immunoturbidimetry or immunonephelemetry) assessments. Five creatinine (MDRD 2006, CKD-EPI 2009 and 2021, EKFC 2021, KRS 2023), two cystatin C (CKD-EPI 2012, EKFC 2023) and three combined eGFR (CKD-EPI 2012 and 2021, combined EKFC) were evaluated. All equations were significantly correlated with mGFR (R² = 0.672–0.745) with a low median bias (+4.2 to −1.1 mL/min/1.73 m²). Chronic kidney disease staging agreements were all above 68% (maximum: 79.3% for CKD-EPI comb 2021). Percentages of eGFR comprised in between 30% of the mGFR ranged from 85.5% to 87.6% (combined equations), from 83.1% to 84.3% (cystatin C equations) and from 75.2% to 81.4% (creatinine equations). Combined creatinine/cystatin C eGFR equations with a P30 value greater to 85% of transplant recipients appeared closer to mGFR than cystatin C or creatinine eGFR.

## Graphical Abstract

**Figure:**
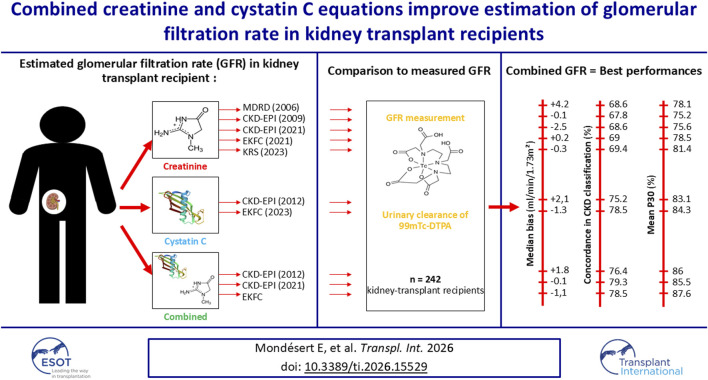

## Introduction

Post transplantation follow-up (PTF) of kidney transplant recipient (KTR) patient recommendations comprises a regular monitoring of estimated glomerular filtration rate (eGFR) [1]. Indeed, longitudinal follow-up of glomerular filtration rate (GFR) could determine transplant trajectories and predict graft failure or improvement of transplant function following adaptation of immunosuppressive treatment [2–5]. Although measured GFR (mGFR) with iohexol or radionuclides permits the most precise GFR assessment, its utilization requires outpatient hospitalization and is often limited to specialized medical structures and thus cannot be used to routinely monitor kidney function [6].

Creatinine is the most used endogenous biomarker to estimate GFR. However, its levels can be influenced by several bias such as muscle mass variation [7] or tubular secretion [8]. KTR patients are particularly concerned by those biases since creatinine tubular secretion tends to increase with kidney function decline and could be impaired by anti-infectious drugs [8, 9]. Moreover, there is a high prevalence of sarcopenia in end stage renal disease patients undergoing hemodialysis [10, 11] and creatinine excretion rate (CER), a direct reflection of muscle mass could be decreased following transplantation [12].

Cystatin C (Cys C) is a complementary endogenous marker used to estimate GFR. Unlike creatinine, it is not secreted in renal tubules and is far less sensitive to muscle mass variations. Cys C have an important place in chronic kidney disease diagnosis and classification as it has been implemented since 2012 in kidney disease-improving global outcome (KDIGO) guidelines [13]. Moreover, the 2024 KDIGO recommendations clearly recommend Cys C use if creatinine estimation could be inaccurate, or if a more accurate assessment of GFR is needed for clinical decision-making or drug dosing [6]. Cys C has also been shown to be a strong predictor of cardiovascular mortality in the general population [14]. Several studies cite data in favor of the use of cystatin C in KTR. [15, 16], but Cys C has also its own bias in this population. For instance, corticosteroid medication that is a commonly prescribed immunosuppressive drug in kidney transplantation tends to increase Cys C concentration [17, 18].

Besides the specific variation factors for each marker, another factor of variability in eGFR is the multiplication of estimation equations (Table 1). Historically, Modification in Renal Diet Disease (MDRD 2006) equation was the first based on creatinine and estimating mGFR [19]. Chronic kidney disease–epidemiology equation based on creatinine (CKD-EPI creatinine 2009) equation had been developed afterwards in 2009 and is probably now the most used worldwide [20]. Nevertheless, the equation had been updated in 2021 (CKD-EPI creatinine 2021) to avoid the “race factor” that was complicated to implement in clinical settings [21]. In 2021, Pottel et al. developed in collaboration with the European kidney function consortium (EKFC) an eGFR equation (EKFC creatinine 2021) using a creatinine rescaling factor to control variation related to age and sex [22]. More recently, another “race-free” creatinine-based eGFR equation was specifically developed in KTR population datasets (KRS 2023), with superior performances compared to others cr-based eGFR equations [23]. Apart from the KRS 2023 equation, the development and validation dataset for eGFR typically includes a small number of KTR patients.

**TABLE 1:** eGFR equations formulas. CREA, creatinine; Cys C, cystatin C; comb, combination.

Equation/Sex [references]	Cr (µmol/L)	Cys (mg/L)	Equation
MDRD 2006 [19]			
Women & men	—	—	175×(CREA/88.4)^−1.154^×Age^−0.203^[×0.742]^a^
CKD-Epi creatinine 2009 [20]			
Women	≤62	—	144×((CREA/88.4)/0.7)^−0.329^ × 0.993^Age^[×1.15]^b^
​	>62	—	144×((CREA/88.4)/0.7)^−1.209^ × 0.993^Age^[×1.15]^b^
Men	≤80	—	141×((CREA/88.4)/0.9)^−0.411^ × 0.993^Age^[×1.14]^b^
​	>80	—	141×((CREA/88.4)/0.9)^−1.209^ × 0.993^Age^[×1.14]^b^
CKD-Epi Cys C 2012 [24]			
Women & men	—	≤0.8	133×(Cys C/0.8)^−0.499^ × 0.996^Age^[×0.932]^a^
Women & men	—	>0.8	133×(Cys C/0.8)^−1.328^ × 0.996^Age^[×0.932]^a^
CKD-Epi comb 2012 [24]			
Women	≤62	≤0.8	130×((CREA/88.4)/0.7)^−0.248^×(Cys C/0.8)^−0.375^ × 0.995^Age^[×1.08]^b^
​	​	>0.8	130×((CREA/88.4)/0.7)^−0.248^×(Cys C/0.8)^−0.711^ × 0.995^Age^[×1.08]^b^
Women	>62	≤0.8	130×((CREA/88.4)/0.7)^−0.601^×(Cys C/0.8)^−0.375^ × 0.995^Age^[×1.08]^b^
​	​	>0.8	130×((CREA/88.4)/0.7)^−0.601^×(Cys C/0.8)^−0.711^ × 0.995^Age^[×1.08]^b^
Men	≤80	≤0.8	135×((CREA/88.4)/0.9)^−0.207^×(Cys C/0.8)^−0.375^ × 0.995^Age^[×1.08]^b^
​	​	>0.8	135×((CREA/88.4)/0.9)^−0.207^×(Cys C/0.8)^−0.711^ × 0.995^Age^[×1.08]^b^
Men	>80	≤0.8	135×((CREA/88.4)/0.9)^−0.601^×(Cys C/0.8)^−0.375^ × 0.995^Age^[×1.08]^b^
​	​	>0.8	135×((CREA/88.4)/0.9)^−0.601^×(Cys C/0.8)^−0.711^ × 0.995^Age^[×1.08]^b^
CKD-Epi creatinine 2021 [21]			
Women	≤61.6	—	142×((CREA/88.4)/0.7)^−0.241^ × 0.9938^Age^×1.012
​	>61.6	—	142×((CREA/88.4)/0.7)^−1.200^ × 0.9938^Age^×1.012
Men	≤79.2	—	142×((CREA/88.4)/0.9)^−0.302^ × 0.9938^Age^×1.012
​	>79.2	—	142×((CREA/88.4)/0.9)^−1.200^ × 0.9938^Age^×1.012
CKD-Epi comb 2021 [21]			
Women	≤62	≤0.8	130×((CREA/88.4)/0.7)^−0.219^×(Cys C/0.8)^−0.323^ × 0.9961^Age^
​	​	>0.8	130×((CREA/88.4)/0.7)^−0.219^×(Cys C/0.8)^−0.778^ × 0.9961^Age^
Women	>62	≤0.8	130×((CREA/88.4)/0.7)^−0.544^×(Cys C/0.8)^−0.323^ × 0.9961^Age^
​	​	>0.8	130×((CREA/88.4)/0.7)^−0.544^×(Cys C/0.8)^−0.778^ × 0.9961^Age^
Men	≤80	≤0.8	135×((CREA/88.4)/0.9)^−0.144^×(Cys C/0.8)^−0.323^ × 0.9961^Age^
​	​	>0.8	135×((CREA/88.4)/0.9)^−0.144^×(Cys C/0.8)^−0.778^ × 0.9961^Age^
Men	>80	≤0.8	135×((CREA/88.4)/0.9)^−0.544^×(Cys C/0.8)^−0.323^ × 0.9961^Age^
​	​	>0.8	135×((CREA/88.4)/0.9)^−0.544^×(Cys C/0.8)^−0.778^ × 0.9961^Age^
EKFC creatinine 2021 [22]			
Women & men	cr/Q ≤1	—	107.3/(CREA/Q)^0.322^×[0.99^Age−40^]^c^
​	cr/Q >1	—	107.3/(CREA/Q)^1.132^×[0.99^Age−40^]^c^
EKFC Cys C 2023 [25]			
Women & men	—	cys/Q ≤1	107.3/(CREA/Q)^0.322^×[0.99^Age−40^]^c^
​	—	cys/Q >1	107.3/(CREA/Q)^1.132^×[0.99^Age−40^]^c^
EKFC comb	​	​	​
Women & men	—	—	(eGFR(EKFC CREA 2021)+eGFR(EKFC Cys C 2023))/2
KRS 2023 [23]			
Women	—	—	e^4.4275492-0.8230475*ln(CREA/88.4)-0.0124264(CREA/88.4)2-0.055068*Age^
Men	—	—	e^4.4275492-0.8230475*ln(CREA/88.4)-0.0124264(CREA/88.4)2-0.055068*Age+0.1806494^

^a^ Factor to apply if the patient is a woman.

^b^ Factor to apply if the patient is African American.

^c^ Factor to apply if age >40 years.

Q = rescaling factor, calculated as follows:

Creatinine: For ages 2–25 years, Women: ln(Q) = 3.080 + 0.177 × Age − 0.223 × ln(Age) − 0.00596 × Age^2^ + 0.0000686 × Age^3^.

Men: ln(Q) = 3.200 + 0.259 × Age − 0.543 × ln(Age) − 0.00763 × Age^2^ + 0.0000790 × Age^3^. For ages >25 years, Women: Q = 62 μmol/L, Men Q = 80 μmol/L.

Cys C: For ages <50 years: Women & men: Q = 0.83 mg/L. For ages >50 years, Q = 0.83 + 0.005*(Age-50) mg/L.

With Cys C use as the endogenous biomarker, the CKD-EPI Cys C 2012 equation was designed like CKD-EPI creatinine 2009 to be calculated with Cys C levels, age and sex [24]. EKFC Cys C 2023 was also designed like EKFC creatinine 2021 but with age as the only factor required to calculate eGFR [25].

Finally, combined equation based on both creatinine and Cys C levels had been developed: combined CKD-EPI 2012 (CKD-EPI comb 2012) which was updated in 2021 (CKD-EPI comb 2021) to overcome the limitation of incorporating a “race factor” into the equation, which is a social construct that can influence medical decisions [21, 24]. The mean of EKFC creatinine 2021 and EKFC Cys C 2023 (EKFC comb) can be also used.

To date, few data comparing all these equations are available in KTR patients. We took advantage of simultaneous mGFR, creatinine and Cys C assessments in KTR patients to evaluate the performances of the ten eGFR equations mentioned above.

## Materials and Methods

### Subjects

This single-center and retrospective study was carried out on patients seen in the Nephrology department of the Montpellier University Hospital between 2007 and 2009. KTR patients aged 18 or more recruited during their normal PTF protocol underwent GFR measurement by urinary clearance of technetium-labelled di-ethylene-triamino-penta acetic acid (^99m^Tc-DTPA). Inclusion criteria comprised concomitant plasma creatinine and serum Cys C measurements and age superior to 18 years. Blood collection took place the same day before any injection and before the start of GFR measurement, whereas 24-hour urine sample was collected the day before GFR measurement. Basic demographical data (age, sex), graft date and medication at the time of mGFR measurement were extracted from medical records, in accordance with approval by our institution local ethics committee declared under the number DC-2008-417.

### Methods

mGFR were measured by urinary clearance of ^99m^Tc-DTPA using the constant infusion technique [26], results were expressed in mL/min/1.73 m^2^, with body surface area calculated by Du Bois and Du Bois formula [27]. For mGFR measurement; four 20–30 min urine collections were obtained by spontaneous voiding after the induction of water diuresis and a 90-min equilibration period. At the end of each clearance period, patients drank a volume of water equal to the preceding urine volume. Radioactivity was determined on the 4 urine collection samples and on 4 plasma samples drawn at midpoint of each clearance period. The measured GFR values was the average of the four calculated clearance. Plasma creatinine assessment using an enzymatic isotope dilution mass spectrometry (IDMS) traceable assay on Olympus® analyzer (Olympus France, Rungis, France) using creatinine reagents from Olympus (Olympus France, Rungis, France) or on architect C8000® (Abott, Chicago, USA) using Abbott enzymatic creatinine method (Multigent® creatinine) was performed. Cys C serum levels were determined by particle-enhanced immunonephelometry (PENIA) using N Latex cys reagents from Siemens on the BNII® systems (Siemens, Marburg, Germany) or by particle-enhanced turbidimetry (PETIA) using Sentinel Diagnostics reagents (Sentinel CH. SpA, Milano, Italy) on Architect C8000® analyzer [15, 28]. BD vacutainer® (Franklin Lakes, New Jersey, United States of America) collection, with lithium heparinate anticoagulation for plasma samples and with clot activator additive (silica particles) for serum samples, were centrifuged at 2000g for 10 min. Creatinine measurements were performed shortly after centrifugation, while serum samples were frozen at −18 °C until analysis performed within 7 days from sampling. When available, CER expressed by urinary Creatinine measured by the same techniques than in the blood on a 24-h urine collection (mmol/24h) preceding mGFR assessment was also collected. eGFR was calculated with the formulas of the equations depicted in Table 1, comprising five CREA-based equations (MDRD 2006, CKD-EPI 2009, CKD-EPI 2021, EKFC creatinine 2021, KRS 2023), two Cys C-based equations (CKD-EPI Cys C 2012, EKFC Cys C 2023) and three combined equations (CKD-EPI comb 2012 and 2021, EKFC comb). For CKD-EPI creatinine 2009 and CKD-EPI comb 2012, the “African-American factor” was not taken into account in eGFR calculation.

### Statistical Analysis

All quantitative values of GFR are expressed in mL/min/1.73 m^2^ and as mean ± standard deviation (SD) in text. Normality was assessed with the Kolgomorov-Smirnov test. Passing-Bablok regression procedure was used to determine the slope (*a*) and the intercept (*b*) of the linear equation eGFR = a*mGFR + *b*. Pearson determination coefficients (R^2^) were determined to compare mGFR with eGFRs, eGFRs based on creatinine or combined eGFRs with 24-hours urinary CREA, eGFRs based on Cys C or combined eGFRs with corticosteroid dosage. Slope tests and t-tests for Pearson correlation were performed with alpha significance level set at 5% and a P-value under 0.05 considered as statistically significant. The scatter of differences was visualized by means of Bland-Altman plot. The concordance between CKD classification according to the 2012 KDIGO definition of patients using mGFR and eGFR was assessed using weighted Cohen’s κ-test coefficient (wκ) with value in between 0.4 and 0.6 indicating moderate agreement, and values in between 0.6 and 0.8 indicating substantial agreement. Concordance was also evaluated by the determination of the proportion of patient with eGFR comprised within ±10% (P_10_) ±30% (P_30_) of mGFR. 95% confidence intervals were calculated for concordance measures. Net reclassification improvement (NRI) was calculated to assess the ability of the best performing eGFR equation (in terms of concordance with mGFR) to correctly classify individuals into CKD stages compared to other eGFR equations with mGFR as the reference standard. Category-based NRI was used with CKD stage cutoffs of 90, 60, 45, 30 and 15 mL/min.1.73 m^2^.

## Results

### Population, eGFR and mGFR

**FIGURE 1:**
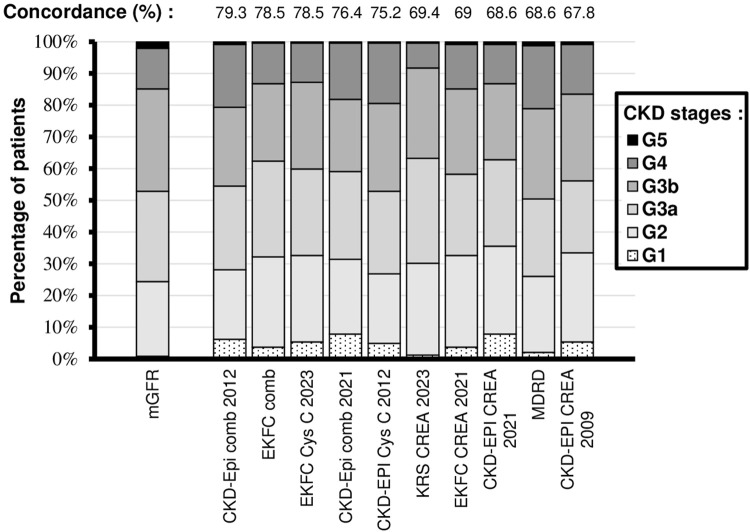
Repartition of mGFR and eGFR values across the cohort. Percentage of concordance in CKD classification with mGFR is indicated for each eGFR equation.

A total of 242 KTR patients (median [min-max] age: 53.2 [18.3–76.6] years, %female/male: 35/65, median ± Interquartile range PTF duration: 2.1 ± 5 years) with simultaneous mGFR, plasma creatinine and serum Cys C measurement were recruited (Table 2). Kolgomorov-Smirnov test showed that CREA, Cys C, mGFR and eGFRs values were normally distributed. Mean mGFR was at 51.4 ± 19.8 mL/min/1.73 m^2^, CKD classifications based on it consisted mainly in G3a (N = 75, 31%) and G3b and G2 (N = 63, 26% for both) stages (Figure 1). Mean creatinine plasma level and Cys C serum level were 144.7 ± 62.3 μmol/L and 1.6 ± 0.6 mg/L respectively. Mean eGFRs ranged from 47.8 ± 19.3 (MDRD) to 54.2 ± 22.2 (CKD-EPI creatinine 2021) mL/min/1.73 m^2^. A poor fit was observed between 24-h urinary creatinine (available for 97% of cases, 234/242 patients) and eGFRs based on creatinine or combined eGFRs (R^2^ = 0.0005 to 0.015, P > 0.05 in every case). The relationship between immunosuppressive corticosteroid dosage (median ± Interquartile range: 5 ± 5 mg, 82% of patients treated) and eGFRs based on Cys C or combined eGFRs was limited in terms of explanatory power (R^2^ = 0.004 to 0.01, P > 0.05 in every case).

**TABLE 2:** Baseline characteristics of the 242 included patients. min, minimum; max, maximum; PTF, post-transplantation follow-up; IQR, interquartile range; SD, standard deviation.

Criteria	Value
Age (years, *median [min-max]*)	53.2 [18.3–76.6]
%Female/ %male (*%*)	35/ 65
PTF duration (years, *median ± IQR*)	2.1 ± 5
^99m^Tc-DTPA mGFR (ml/min/1.73 m^2^, *mean±SD*)	51.4 ± 19.8
CKD classification based on mGFR (*%*)	
G1 (>90 mL/min/1.73 m^2^)	3.3
G2 (60–90 mL/min/1.73 m^2^)	26
G3a (45–60 mL/min/1.73 m^2^)	31
G3b (30–45 mL/min/1.73 m^2^)	26
G4 (15–30 mL/min/1.73 m^2^)	11.6
G5 (<15 mL/min/1.73 m^2^)	2.1
Creatinine (µmol/L, *mean±SD*)	144.7 ± 62.3
Cystatin C (mg/L, *mean±SD*)	1.6 ± 0.6
Mean eGFR (ml/min/1.73 m^2^, *mean±SD*)	
MDRD 2006	47.8 ± 19.3
CKD-Epi CREA 2009	51.6 ± 21.5
CKD-Epi CREA 2021	54.2 ± 22.3
EKFC CREA 2021	51.5 ± 20.3
KRS 2023	51.2 ± 16.2
CKD-Epi Cys C 2012	49.4 ± 21.5
EKFC Cys C 2023	52.8 ± 20
CKD-Epi comb 2012	49.4 ± 21
CKD-Epi comb 2021	51.8 ± 22
EKFC comb	52.2 ± 19.4

### Regression, Correlation, Bias and Concordance Between mGFR and eGFR

**FIGURE 2:**
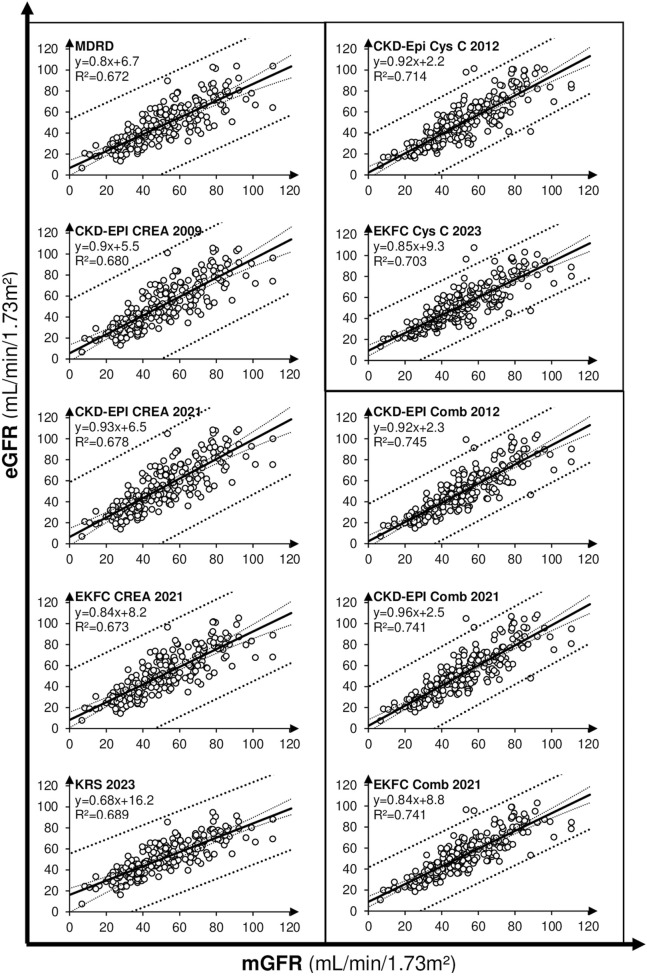
Regression between mGFR (x axis, mL/min/1.73 m^2^) and eGFRs (y axis, mL/min/1.73 m^2^). Creatinine equation are presented in left, Cys C and Comb equations in right.

Regression and correlation between mGFR and the 10 tested equations are shown in Figure 2 and values are summarized in Table 3. A statistically significant relationship with a slope test P-value <0.001 was found for all equations. Passing-Bablok regression yielded slopes values comprised between 0.8 and 0.96 for all equations except for KRS which was substantially lower (0.68). KRS equation presented also the highest intercept (16.2), while CKD-EPI Cys C and combined equations presented the lowest intercepts (2.2–2.5). Only three eGFRs (CKD-EPI Cys C, Comb 2012 and 2021) did not present a constant error compared to mGFR as 95% confidence intervals for the intercept included zero. On the other hand, only CKD-Epi creatinine 2021 and CKD-EPI Comb 2021 had a 95% confidence interval of the linear equation eGFR = a*mGFR + *b* slope (*a*) comprising one, indicating the absence of proportional error. R^2^ coefficients were higher for combined equations (0.741–0.745) compared to Cys C (0.703–0.714) and creatinine ones (0.672–0.689). T-tests for Pearson correlation resulted in a statistically significant correlation (P < 0.001) between each eGFR and mGFR.

**TABLE 3:** mGFR-eGFR equations correlation, regression and agreement measures with 95% confidence intervals. Passing-Bablok regression procedure fits the parameters *a* and *b* of the linear equation eGFR = a*mGFR + *b*. CREA, creatinine; Cys C, cystatin C; comb, combination.

Equation	R^2^	Slope (*a*)	Intercept (*b*)	Cohen wκ	Concordance in CKD classification (%)	Mean P30 (%)
MDRD 2006	0.672	0.80 [0.73, 0.87]	6.7 [2.8, 10.6]	0.566 [0.615, 0.517]	68.6 [62.7, 74.4]	78.1 [72.9, 83.3]
CKD-Epi CREA 2009	0.680	0.90 [0.82, 0.98]	5.5 [1.2, 9.8]	0.550 [0.600, 0.500]	67.8 [61.9, 73.7]	75.2 [69.8, 80.6]
CKD-Epi CREA 2021	0.678	0.93 [0.85, 1.01]	6.5 [2.0, 11.0]	0.546 [0.596, 0.496]	68.6 [62.7, 74.4]	75.6 [70.2, 81]
EKFC CREA 2021	0.673	0.84 [0.77, 0.92]	8.2 [4.1, 12.3]	0.552 [0.602, 0.502]	69 [63.2, 74.8]	78.5 [73.3, 83.7]
KRS 2023	0.698	0.68 [0.62, 0.74]	16.2 [13.0, 19.4]	0.515 [0.567, 0.463]	69.4 [63.6, 75.2]	81.4 [76.5, 86.3]
CKD-Epi Cys C 2012	0.714	0.92 [0.84, 0.99]	2.2 [−1.9, 6.2]	0.649 [0.696, 0.602]	75.2 [69.8, 80.6]	83.1 [78.3, 87.8]
EKFC Cys C 2023	0.703	0.85 [0.78, 0.92]	9.3 [5.4, 13.1]	0.594 [0.643, 0.545]	78.5 [73.3, 83.7]	84.3 [79.7, 88.9]
CKD-Epi comb 2012	0.745	0.92 [0.85, 0.98]	2.3 [−1.5, 6.1]	0.675 [0.720, 0.630]	76.4 [71.1, 81.8]	86 [81.6, 90.3]
CKD-Epi comb 2021	0.741	0.96 [0.89, 1.03]	2.5 [−1.4, 6.5]	0.712 [0.755, 0.669]	79.3 [74.2, 84.4]	85.5 [81.1, 90]
EKFC comb	0.741	0.84 [0.78, 0.91]	8.8 [5.3, 12.3]	0.686 [0.731, 0.641]	78.5 [73.3, 83.7]	87.6 [83.5, 91.8]

**FIGURE 3:**
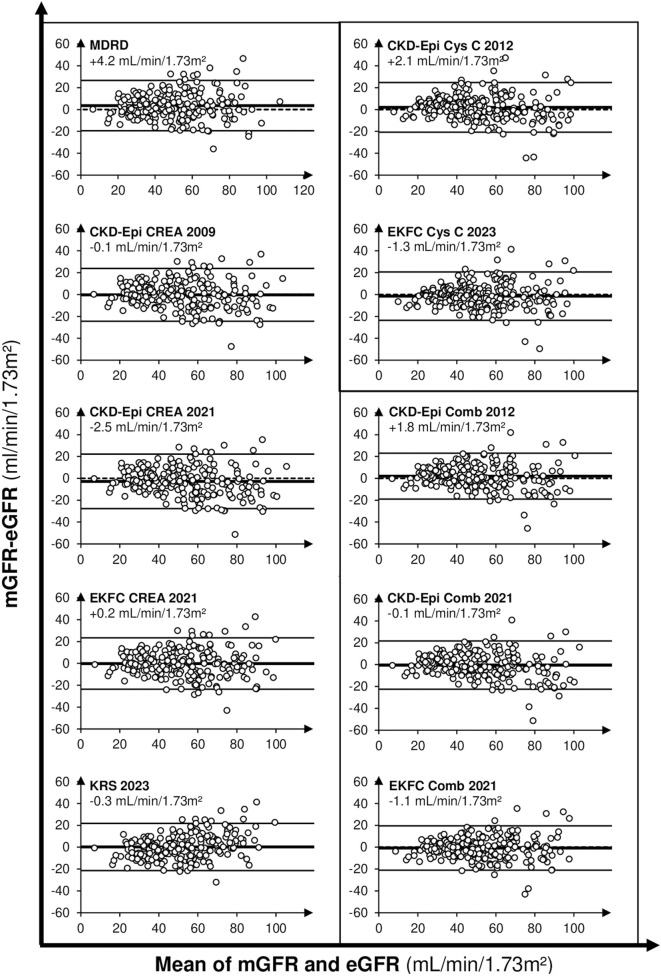
Bland-Altman’s plots between mGFR and eGFR. x axis represents mean of mGFR and eGFR (both in mL/min/1.73 m^2^), y axis represents the difference between mGFR and eGFR. Creatinine equation are presented in left, Cys C and Comb equations in right. Median bias between mGFR and eGFR are indicated for each equation.

All equations presented a median bias (i.e., mGFR-eGFR) comprised in between ±2 mL/min/1.73 m^2^ compared to mGFR except for MDRD median bias, underestimating mGFR by 4.2 mL/min/1.73 m^2^ on average. Scatter of differences visualized on Bland-Altman plots (Figure 3) showed an equally positive and negative repartition, with a higher imprecision of eGFR when mGFR values are increasing.

Agreements measures are listed in Table 3. Overall, combined equations gave the best performances in terms of percentages of agreement in CKD classification, wκ index and P_30_ values followed by Cys C equations and then by creatinine equations. CKD-EPI comb 2021 had the highest percentage of agreement in CKD classification (79%) and highest wκ (0.712) indicating a substantial agreement. Substantial agreement was also noted for other combined equations and CKD-EPI cys 2012, whilst a moderate agreement was obtained for all other equations (wκ values from 0.515 to 0.594). P_30_ value was the highest for EKFC comb with 88%.

Using CKD-EPI comb 2021 as the reference eGFR equation, nine alternative eGFR equations were evaluated using CKD stages category-based NRI relative to mGFR (Table 4). A positive NRI was observed for 8 out of 9 equations (from 2.5 for EKFC Cys C to 12.4 for CKD-Epi CREA 2021), indicating a superior CKD classification ability of CKD-EPI comb 2021. A negative NRI was observed only for EKFC comb (−2.48).

**TABLE 4:** Net reclassification improvement (NRI) of eGFR CKD-Epi comb 2021 to correctly classify individuals into CKD stages compared to eGFR calculated with other equations with mGFR as the reference standard.

Equation	NRI
MDRD 2006 CKD-Epi CREA 2009 CKD-Epi CREA 2021 EKFC CREA 2021 KRS 2023 CKD-Epi Cys C 2012 EKFC Cys C 2023 CKD-Epi comb 2012 EKFC comb	10.3 9.5 12.4 10.7 7.9 6.6 2.5 1.7 −2.5

### P_30_ and Bias According to Age

**FIGURE 4:**
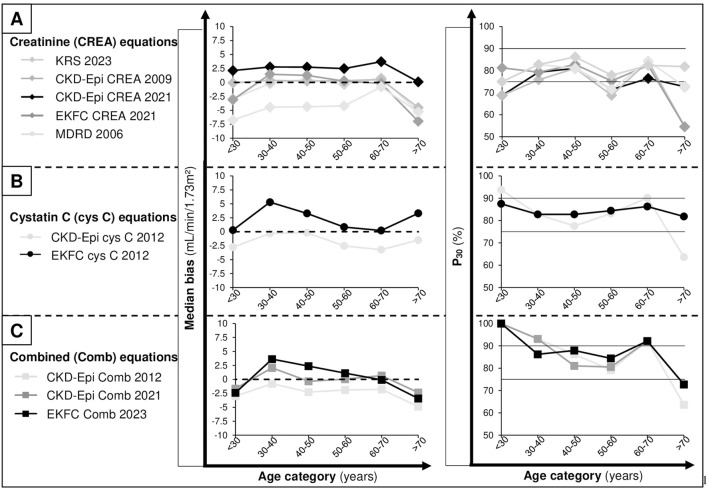
Estimation equation performances in terms of median bias (left panel) and P_30_ (right panel) according to different ages categories. **(A)** Creatinine (CREA) equations performances. **(B)** Cystatin c (cys C) equations performances. **(C)** Combined (comb) equations.

Figure 4 shows the P_30_ and median bias according to age categories. Cys C and combined equations appeared to systematically underestimating mGFR for patients younger than 30 years, in contrast with patients aged 30–40 years where a systematic overestimation was observed. Creatinine equations presented more variable bias repartition, with CKD-EPI 2009 having the less important bias and MDRD the worst. In addition, a systematic underestimation of mGFR by all creatinine equations was noted in the patients aged over 70 years.

All equations presented a mean P_30_ greater than 75% for patient <70 years old which has been considered “sufficient for good clinical decision making” by the Kidney Disease Outcomes Quality Initiative [29]. However for older patients (>70 years-old) only EKFC Cys C and KRS creatinine remained around 80%, all other equations fell below 75%. Combined equations had the highest rates of P30 with some values greater than 90% which is the goal for optimal clinical decision making [29], except for patients aged above 70 years.

## Discussion

The present study was designed in order to determine the best biomarker/equation combination for eGFR determination in KTR settings. Our results clearly indicate that combined equations have better performances than Cys C and creatinine eGFR-based equations in terms of correlation and bias (Figures 2–4) with reference method and in terms of concordance performances for CKD classification (Table 3). CKD-Epi comb 2021 was notably the best performing equation when looking at Passing-Bablok regression (eGFR = a*mGFR + *b* equation), being the only equation having slope (*a*) interval confidence including one (0.89–1.03) and intercept (*b*) confidence interval including zero (−1.4-6.5). Cys C is however not implemented in all laboratories yet and therefore the use of EKFC creatinine 2021 or CKD-EPI creatinine 2021 over CKD-EPI creatinine 2009 may be recommended because the “race factor” is not needed and the equations are simplest to calculate. Moreover, CKD-Epi comb 2021 showed a positive NRI of CKD classifications compared to most of the other equations with mGFR as a reference, only EKFC comb performing slightly better (Table 4).

Accurate GFR estimation is of critical importance for KTR patient, since eGFR trajectory changes can lead to important clinical decisions like immunosuppressive treatment management, dialysis reinstauration or reinscription in kidney transplantation list [1–5]. Gold standard measurement methods should be performed to have the most precise GFR determination, but cannot be routinely implemented in KTR patient follow-up [6]. Prompt GFR estimation with the help of endogenous markers and adequate equation is therefore advisable. KDIGO guidelines for KTR patient recommend a regular blood creatinine measurement for eGFR determination in PTF, at an increased rate the year after transplantation and every 2–3 months after the first year post-transplantation [1]. However, no precise eGFR formula is recommended in the guideline, and at the time of publication of the guideline (2009), only MDRD 2006 was available. Since then, multiplication of published equations has been observed (Table 1), and Cys C is being more widely used. eGFR based on blood Cys C assessment is notably included in the algorithm of eGFR initial assessment of the recent 2024 KDIGO guideline [6]. Numerous eGFR equations and cr/cys bias in the specific KTR population can lead to a substantial variability of eGFR results.

Interestingly, in our cohort CREA-based equations appeared to underestimate mGFR in older patients. A possible explanation could be the high prevalence of sarcopenia in this age category [10, 11]. However, no correlation was found between daily urinary excretion of creatinine and plasma creatinine or eGFR based on creatinine. On the counterpart, Cys C appeared to underestimate mGFR in younger patients aged under 30 and overestimate mGFR in patients aged between 30 and 40 years, without any clear explanation. Nonetheless, Cys C levels can be influenced notably by high corticosteroids dosages that can be encountered in transplantation for immunosuppression purposes [17, 18] but we did not find any correlation between Cys C or eGFR Cys C and corticosteroids dosages. Combined equations seem to limit those specific biomarker bias by taking into account another unbiased biomarker. Interestingly, a previous article demonstrated that a bias exists between eGFR based on Cys C and eGFR based on creatinine in KTR patient [30]. Moreover, it has been showed that GFR based on creatinine is more accurate than eGFR based on Cys C for determining associations with clinical outcomes due to low GFR when mGFR is not available [31]. This finding is consistent with the results published by Pottel and colleagues [22] where combined equations presented the best P_30_ values across ages in a general population of 12,832 patients. Also consistent with our results, Meeusun and colleagues showed as well that CKD-EPI comb 2012 performed better than CKD-EPI creatinine 2009 and CKD-EPI Cys C 2012 in a population of 568 KTR patients [32].

Regarding equation diversity, no equation seems to outperform another one in term of agreement when looking in each biomarker group. In term of absolute bias, no equation surpasses 3 mL/min/1.73 m^2^ with the exception of MDRD 2006 equation with a median bias of +4.2 mL/min/1.73 m^2^. This latter can lead to important errors and MDRD 2006 should therefore not be used in KTR patient.

In order to prevent some regional heterogeneity on creatinine level and eGFR estimation [25, 33], we tested the EKFC equations in our monocentric cohort. By contrast to previous reports [25], we did not observe a clear difference with the CKD-EPI equations. EKFC equation is being constructed with a Q-rescaling factor representing concentration of serum creatinine or Cys C in healthy males and females. Population-specific Q-rescaling factor have been proposed [34] providing remarkable performances in those populations. A specific Q-rescaling factor for KTR patients is maybe necessary to improve EKFC performances in this specific context.

Moreover, the KRS equation developed for KTR patient was not superior to other CREA-based equations. The overall P_30_ (81%) of KRS equation was lower of the overall P_30_ (90%) obtained in the development cohort [23]. Additionally, this equation had much worst Passing-Bablok regression results (eGFR = 0.68*mGFR+16.2) compared to all other equations, which could compromise his use.

We acknowledge that this study suffers from limitations: a limited patient number, a monocentric study setting and a variability of creatinine and Cys C employed assays. Nevertheless, all creatinine assays were based on a standardized enzymatic method as recommended by guidelines [35]. Regarding Cys C, measurements were realized before the establishment of ERM-DA471/IFCC reference material that have proven to improve performances of Cys c assays [36]. However, switch ability between the two PENIA and PETIA assays used in the present study were tested in a previous study [28]. Both assays yielded very close results on repeated measurements of five pooled serum samples with cystatin C values ranging from 0.6 to 1.4 mg/L. Furthermore, the Siemens PENIA assay was tested and showed good correlation with both IDMS reference method [37] and with many other PETIA assays [15, 38, 39]. The use of urinary clearance of ^99m^Tc-DTPA method for mGFR determination is also a limitation because eGFR equations are generally build on other mGFR determination methods such as iotholamate for CKD-Epi and inulin, iohexol clearance and radionuclide clearances including 99mTc-DTPA for EKFC. Comparability between mGFR methods is often not observed, and steps towards standardization are needed [40, 41].

In conclusion, the conjoint use of creatinine and Cys C with combined equations in KTR patient follow-up offers the best performance in estimating GFR in our experience. Further studies on larger cohorts need to be conducted to confirm these results.

## Data Availability

The raw data supporting the conclusions of this article will be made available by the authors, without undue reservation.
